# Bibliographical Notices

**Published:** 1847-05

**Authors:** 


### BIBLIOGRAPHICAL NOTICES.

*Six Lectures on the Uses of the Lungs; and causes, prevention and cure of Pulmonary Consumption, Asthma, and Diseases of the Heart; on the laws of longevity, and on the mode of preserving male and female health to an hundred years. With twenty-eight illustrations.* By SAMUEL SHELDON FITCH, A. M., M. D. 12mo., pp. 324. New York, 1847.

We take up the notice of this production with feelings of contempt, not unmixed, however, with sorrow. Nearly twenty years have elapsed since its author published "A system of Dental Surgery." He was then a reputable dentist; holding a position amongst the best of the "craft;" and his "system" was esteemed so creditable to him in all respects, that it obtained encomiums from those whose good opinion it must have been his pride to possess. And now—*quantum mutatus!* The *auri sacra fames* has, we fear, induced him to quit the path of credit; to abuse the profession of which he is an enrolled member; and in the production before us to exhibit an amount of ignorance, both on general and medical topics, which must excite the wonder of every one how he could have been entitled to the suffixes of A. M. and M. D., which he attaches to his name.

We are apt to despise the miserable mercenary, who, without any claim to medical attainments, endeavours to gain a wretched subsistence on the gullibility of his brethren; but what ought to be our feelings, when a licensed member of the profession quits its honest paths, and wanders into the devious wilds of empiricism. Nor is the author of these lectures the only one to whom these remarks apply. We have reason, indeed, to believe, that the *fancied* success of others, a few years ago, led him to turn his attention to this subject; and that bromine was to be one of the hacks on which he designed to ride into notoriety and pelf. The example of Ramadge—the great archetype for the smaller fry that have succeeded him—himself belonging to the Royal College of Physicians of London; and yet a greater empiric perhaps hardly exists, as we had occasion to hint when noticing the translation of Läennec, with which his name has been connected—has led to a host of inhalers and consumption curers, who not only blazon amongst the public their own false or fancied [?] statements of success, but obtain from men in the ranks of the profession—as we shall mention more particularly hereafter—testimonials for the furnishing of which—if they have a spark of interest remaining in their noble calling—they must, by this time, be heartily ashamed.

The "Lectures" before us are evidently addressed to the people, not to the profession, whose members, indeed, they grossly slander and abuse. This fact, as well as the meretricious title, would have prevented us from taking the slightest notice of them, were it not that the "A. M., M. D." might mislead some of our readers, and induce them, in their want of information, to send for the work. We cannot refer to all the examples of gross ignorance and misrepresentation that pervade it; but it may be well to cite a few, with the view of showing, that these charges against the author and his production are not only sustained, but that our expressions have been even too guarded. "In 1827," he remarks, "whilst pursuing some investigations in Philadelphia upon nervous influence, preparatory to my graduation thesis, I discovered what I conceive to be the grand uses of the lungs, and their first great purpose. In these researches I was led to ask, what it was that gave support and power to the nervous system. I traced this support to the lungs; and at once and forever, to my mind, all darkness upon the uses of the lungs disappeared." p. 28. And what does the reader suppose was the essence of this immortal "discovery"?—that the lungs "give to the human machine its power of action. This power exists in the atmospheric air; and the lungs are the medium by which, and through which, that principle which gives the human machine its living power is conveyed to it." This immediately accounted to him for the fact that women are weaker than men. "The lungs of women are one-third smaller than those of men;"—and why one man is stronger than another. "Were you required to select the strongest man of your acquaintance, would you select a man with a flat, thin chest, long neck, and narrow, round, stooping shoulders, or would you select a man with a wide, deep, round chest, and broad, heavy shoulders? There can be but one answer. You would choose the man with large lungs, and you would not be disappointed. You would find his strength in the exact ratio of his pulmonary developement, other things being equal." It explained to him also, satisfactorily, why we use the right arm rather than the left. "It is seen with all the natives of this globe, that the right arm is preferred in its use [?] over the left; in other words, that all men are right-handed, as a general rule. Some very unsatisfactory reasons are given for this. The true reason is found in the fact that the lungs give us the power of action, and that the right lung is larger than the left; hence it gives more power to the right arm. I have often seen the right arm hang quite useless at the side by extensive disease of the right lung. [!] Very rarely we find persons left-handed. I believe in all cases where they are left-handed, the left lung will be found the largest. I have often had an opportunity of verifying this fact; so that being left handed is not a matter of capricious or accidental choice in the infant, but is owing to the left arm being the strongest, because the left lung is the largest." p. 31.

But there is no end to the removal of difficulties effected by this "discovery." It explains, for example, why a brother in active life in the country possesses "in a vast many cases double the physical strength of the brother in the city,"—the lungs of the rustic brother being, we presume, larger:—why the eagle "that poises himself high in the heavens, and, almost with the rapidity of lightning sweeps to the earth, and seizing a living animal of nearly or quite his own weight, flies away with him to the top of Mont Blanc;"—why "many [qr. how many?] migratory pigeons that travel fifty miles an hour, that you can hold upon your hand, [qu. the pigeons, ?] consume more air than some females:"—but we pause for breath; our lungs are not sufficiently capacious for the "power," the muscular exertion needed to record all the ramifications of this great "discovery."

Next follow some remarks—not very connected, it is true—on those wanderings from the true path by men of science, which show that in too many cases "they have started wrong; that their premises are erroneous." As, for example, the "hieroglyphics of Egypt;"—by which the author, be it observed—does not mean to convey, that the hieroglyphics "have started wrong, or that their [the hieroglyphics'] premises are erroneous;" but that by a discovery analogous to his own, "we now learn that what *was* supposed to be a cloud, covering most inscrutable mysteries, *are* only simple records of men's actions, wishes, lives and deaths, and *were* once familiarly read by all the stone-masons of Egypt." Then comes the application of this to the lung-discovery. "A perfect cloud" rests over the lungs, their uses and diseases. "Their grand purpose is entirely overlooked, and unknown by the mass of medical and other men:"—and now follow sentiments, which, if the "discovery" had not been made, ought still to have been sufficient to immortalize the author! "I do not know that I ever met two physicians who were of any eminence and not mere imitators, who thought or acted alike in managing consumption, or who had the least notion how it might be prevented. Nor have they any confidence in their own practice: in few cases have they the least hope of curing the disease or preventing it." * * * "Intimate to the medical body, that consumption is a curable disease, and at once such an idea is denounced as the height of folly or knavery. What results from this darkness of the Faculty? [of which, be it observed, he still professes, but we hope is not considered, to be one.] Why the whole land is covered with a pall; nearly one-half of the adults, when they die, die of consumption or diseases of the chest. The whole population are running everywhere for aid. All confidence in the regular medical faculty for consumption, is lost; nobody respects them, and they do not respect themselves on this subject;"—and then comes the climax, the apotheosis! "Allow me here to say, from a vast experience [!!] that nine-tenths of all that is laid down in medical books, taught in medical schools, or pursued in medical practice, for the prevention and cure of consumption, is calculated to make the disease, not to cure it." And again, after referring to the published opinions of Dr. Chapman, who, he says, "solemnly declares that in a practice of fifty years he has never seen a case of seated consumption cured;"—but who, we beg leave to inform the author, with all due deference to Dr. Chapman, is very far from being the exponent of the views on this subject of the most enlightened pathologists—he exclaims, in tone, and *English*, in entire keeping with the rest of his observations: "This, gentlemen and ladies, is a record of his 'practice,' and of nearly all, with scarcely an exception, in the regular faculty up to this time; and most fully confirms all I have said upon the awful destructiveness of their practice. This leads me also to call to your minds, that nearly all the higher classes in this country, who only employ the old school physicians, when struck with consumption, die of it. Whilst with the more independent and thinking classes, not trammelled by fashion; not reverencing mere names and pretensions, but fly from such persons, and ask for facts, demand cures, find often, at last, in perhaps an obscure old woman, or some illiterate person, that aid which could not be obtained from their regular, and 'world and time-honoured physicians.' This want of the success of the school-bred physicians is owing to their profound ignorance of the uses of the lungs. Why do they not at once, as honest men, tell their consumptive patients, we cannot cure you, and leave them to nature and its resources, without adding to their sufferings the accumulated and accumulating ills of drugs and medical remedies, that, in nine cases out of ten, hurry them to their graves, and deprive them of all comfort whilst living." p. 36.

Yet it must be gratifying to the profession to learn, that the author says all this for the sake of truth, and that he is full of loving kindness for them. "My father, my grandfather and brother," [how are the mighty fallen !] "were regular, eminent, and respectably educated physicians. No one respects them [the profession] more, but I do deplore their ignorance of the uses of the lungs, and causes of consumption ; and most of all, their ignorance of even rational practice in pulmonary diseases." p. 36.

But we need proceed no farther. Are we not then justified in the terms of reprehension in which we have spoken of these "Lectures ;" and can we be surprised at the declaration of the author, that besides his opportunities in London, he has had *five thousand consultations* in cases of consumption and kindred diseases within the last three years,"—that is *fourteen new cases* daily, and every day during that time !—and that in no instance has he ever had a decision of his proved incorrect by any physician ? Nor will any one be astonished, that advertisements, of the most nauseous character, have been left at the doors of respectable dwellings in this city, filled with, perhaps, in many cases *bought* testimonials in favour of the "lectures." We say, "perhaps in many cases bought;" for the editors of a respectable and independent journal in a neighboring city have affirmed, that they were offered a sum of money for a favourable notice,—that they spurned the offer,—and noticed the paltry production in terms that it richly merited.

One word more. Certain physicians have furnished testimonials to the author of these "Lectures," and as they have courted—what we, however, consider unenviable—notoriety, it is proper that they should enjoy it. Dr. Luther Brigham, of Lowell, introduces him as "justly celebrated for his researches in the uses of the Lungs, and the nature and treatment of Pulmonary Consumption. I think his opinion and advice on these subjects of great value. His patients in this place speak of him in the highest terms." Dr. Benjamin West, of Nantucket, speaks of him as "one who is acquainted, to a *surprising* extent, with the subject (consumption) in its most important bearings; and who by his disinterested actions has shown himself entitled to the respect and confidence of all with whom he may be brought in contact." Next we have a letter from Dr. Hubbard Graves, of Nashville, N. H., introducing him to a professional brother as his "friend" Dr. Fitch, who had recently delivered some lectures on consumption, its causes and cure, in that place. "On my announcing his subject," he says, "the idea of quackery may possibly strik eyou, but there you will be most agreeably disappointed. Dr. Fitch regularly studied his profession, both in this country and in Europe; and you will find him a man of strictly philosophical mind, who has thoroughly examined the theory which he advances. His ideas are not crude and confused as those of quack-lecturers invariably are; you will find that they are clearly arranged, and that all his conclusions have been logically deduced. In fact, from what I have seen of Dr. Fitch, I am satisfied you will deem his acquaintance in the highest degree agreeable."

Messrs. Brigham, West, and Graves, have acted as gentlemen ushers and prolegomena to their "friend," and they will doubtless pride themselves—for "*nulla vestigia retrorsum*"—in having manfully aided to diffuse the new light—the "discovery;"

and to obtain some of the effulgence reflected from the luminary to which they have consented to act as satellites; and in the language of Pope, it may be asked

"Why Jove's satellites are less than Jove?"

They cannot therefore complain, that they are all placed by us in the same category, or that the maxim *noscitur a sociis* should apply to each of them.

---

*Lectures on the Comparative Anatomy and Physiology of the Vertebrate Animals, delivered at the Royal College of Surgeons of England, in* 1844 *and* 1846. By RICHARD OWEN, F. R. S., Hunterian Professor, and Conservator of the Museum of the College. *Part* I. *Fishes. Illustrated by numerous Wood cuts.* 8vo. pp. 308. London, 1847.

We turn with pleasure from the ungracious task of impaling ignorance and unblushing empiricism, to notice the labours of one who has attained a well earned and well merited preeminence in certain departments of medical science more especially. Perhaps at this day, indeed, no name stands higher in the roll of living comparative anatomists than that of Richard Owen,—a successor of the amiable and excellent Cliff, who filled so ably the office of Conservator of the Museum of the Royal College of Surgeons, of London,—from its first establishment, if we mistake not.

The volume before us is the second of the series,—the first having investigated the comparative anatomy and physiology of invertebrate animals; and a third is to follow, devoted to the consideration of the mammalia in the same relations.

We cannot do more than say, that after an "introductory lecture" on the characters of the classes of vertebrate animals, the work examines, in succession, the osteology, myology, neurology, digestive system, vascular system, pneumatic and renal organs, and generative system of fishes, in a manner so able and perspicuous as to make us the more anxious to possess the third and remaining volume. When completed, it will form a *thesaurus* on the subjects embraced by it, which every scientific inquirer into the animal economy ought to hasten to possess.

*New Elements of Operative Surgery.* By ALF. A. L. M. VELPEAU, Professor of Surgical Clinique of the Faculty of Medicine of Paris, etc. etc. etc. *Carefully revised, entirely remodelled, and augmented with a treatise on Minor Surgery. Illustrated by over* 200 *engravings, incorporated with the text; accompanied with an atlas in Quarto of twenty-two plates, representing the principal operative processes, surgical instruments, &c. First American, from the last Paris edition.* Translated by P. S. TOWNSEND, M. D., Late Physician to the Seaman's Retreat, Staten Island, New York. *Augmented by the addition of several hundred pages of entirely new matter, comprising all the latest improvements and discoveries in Surgery, in America and Europe, up to the present time. Under the supervision of, and with notes and observations by,* VALENTINE MOTT, M. D. Professor of Surgery with Surgical and Pathological Anatomy in the University of New York; etc. etc. etc. In three volumes. New York: Samuel S. and William Wood, 1847.

The appearance of the *first* and *second* volumes of this important work was duly chronicled as they successively came from the press; and now we have to announce the *third* volume, with the accompanying atlas, which completes the publication. We congratulate Dr. Townsend on the conclusion of his arduous task, for such it unquestionably has been. To translate, collate, and prepare for the press three such huge volumes is no trifling matter; and to have carried the work through without interruption or delay is in the highest degree creditable to his zeal and industry. It must be confessed, however, that the haste with which this has been accomplished has not been without some disadvantages. All who have occasion to examine the volumes will find reasons enough to regret that a little more time was not given to arrangement of the subjects of which they treat, as well as condensation of the language in which the various topics are discussed. Of course we allude to the *American* portion. The volumes might in this way have been materially lessened, much useless detail avoided, and greater perspicuity of expression attained. The faults in style, although not an *essential* matter, are such, in many instances, as ought certainly to have been avoided by so practised a writer as the Editor. What apology, for instance, can be offered for such Gallicisms as "M. S. Cooper" (Mr. Samuel Cooper), "M. Dudley," "M. Kirby," "M. Bostock," "M. Fahnestock," &c. If it be allowable at all to render the names of Frenchmen thus, there surely can be no reason, in an English version, for translating English names into French. The habit of the Editor, of using the pronoun in the first person plural, as is the custom of journalists, instead of the first or third person singular, when uttering his own sentiments, is hardly consistent with good usage. But in the notes by Dr. Mott, the singular and plural form of the pronoun are used indiscriminately. Such sentences as the following, for instance, are common: "The means which I have resorted to, may possibly have been used by others before my time, but I am not aware of the fact. We are sure that many of the cases we have met with, had been abandoned," etc. Such jumbling of words is not only inelegant, but tends to confuse the reader, and the editor's privilege should have been exercised for their correction. But, notwithstanding these and many even greater imperfections, the publication is one of very great worth, and the profession is under lasting obligations to Dr. Townsend for the service he has rendered to Surgical Science in putting this work of Velpeau in an English dress; and to Dr. Mott for the many valuable contributions from his own extensive experience with which this edition is enriched.

---

*A Practical Treatise on the Diseases of Children.* By D. Francis Condie, M. D., Secretary of the College of Physicians; Member of the American Philosophical Society; Honorary Member of the Philadelphia Medical Society, etc. Second Edition, revised and improved. 8vo. pp. 657. Lea & Blanchard. Philadelphia, 1847.

On the appearance of the first edition of this work, we pronounced it to be the best treatise on the diseases of children in the English language; and notwithstanding all that has been published since, we still regard it in that light.

"In the preparation of a new edition of the present treatise," says the author in his preface, "every part of the work has been subjected to a careful revision; several portions have been entirely re-written; while, throughout, numerous additions have been made, comprising all the more important facts, in reference to the nature, diagnosis, and treatment of the diseases of infancy and childhood, that have been developed since the appearance of the first edition."

The first *part* treats at length and with great clearness of the "management of children," of their "peculiarities of organization," the "pathology of infancy and childhood," semeiology," etc., and is alone worth the price of the book. The subjects which appear more particularly to have undergone revision, as far as a hasty examination has allowed us to discover, are, pleurisy, pneumonia, croup, and tubercular meningitis; and of the eruptive diseases, measles and scarlatina. On these, as well as the various other subjects embraced in the work, the author has evidently read much and observed well, and his remarks may be fairly admitted as containing the most approved doctrines of the day. His pathological views we regard as particularly sound; and, generally speaking, we find little to object to in his therapeutics, although we cannot always agree with him in the means he employs to give them effect. His attention to German medical literature is discoverable in his fondness for what may be called German prescriptions, or German remedies; thus the chlorohydrate of ammonia in visceral inflammations and cutaneous diseases; and hyoscyamus, variously combined, with ipecacuanha, mild chloride of mercury, prepared chalk, etc., for a wonderful variety of affections. To the formulæ throughout the book we have found more occasions for exception than any thing else. Occasionally, too, we have examples of medicines miscalled, such as "*hydrochloride of ammonia*," etc. In a work from a less competent author, or one of less general excellence, we should not think of naming such imperfections, but in the present instance we feel jealous that any of the kind should exist. There is one point, however, in which our experience differs so greatly from that of the author, that we cannot refrain from noticing it—it is in the quantity of the watery extract of opium which may be safely given to children. At page 335, treating of hooping cough, we have in a note the following prescription and direction for its administration.

℞. Opii Pulv. ℨj.

Aq. Bullient. ℥iij.

Let it stand for three hours, then strain, and add ℨj. bi-carbonate of soda. Dose, for a child two years old, a teaspoonful every three hours.

If given according to the direction above, a child two years old would take the watery solution of *twenty grains of opium* in twenty-four hours.

What effect hooping cough may have in modifying the susceptibility of the constitution to the action of the remedy, or how far the alkali may modify its narcotic properties, we are not prepared to say; but notwithstanding our very great confidence in the experience of the author, the dose is certainly much greater than we could venture to administer to so young a subject.

We cannot close this notice without again expressing our high estimate of the work. The present edition of it, as the author has promised, has been carefully revised, and presents "a full and connected view of the actual state of the pathology and therapeutics of those affections which most usually occur between birth and puberty."

---

CHARGE *to the Graduates of Jefferson Medical College of Philadelphia.* Delivered March 25th, 1847, by Professor DUNGLISON. With a list of the Graduates. Published by the Graduating Class.

We cannot better express our own opinion of this excellent address, than by quoting the language of the able and courteous editor of the New York Annalist. "Dr. Dunglison's address is, as might be expected from so ripe and rare a scholar, highly appropriate. Good," [and Godman] "in our own, and Park, in a sister profession are referred to as showing what can be done in the way of acquiring solid accomplishments, by enduring perseverance at the commencement of a professional career. Study, courtesy, caution, honor, &c., are all, as becomes a gentleman and a conscientious teacher, forcibly recommended to the medical *debutante*, and an eloquent and feeling peroration dismisses to the active duties of their calling, 'the largest class of graduates that has ever graced the halls of Jefferson Medical College, or of any similar institution in the country.'"

The following extract from the address, indicates the sources from whence the large classes of the College are drawn. Few of those who are not immediately connected with the medical institutions of Philadelphia, have an idea of the great distances from whence medical students are attracted to this city.

"You are here from regions widely distant from each other—not from 'Greenland's icy mountains,' but from 'India's coral strand'—for one of you is from remote Burmah. Representatives, too, are amongst you from Ireland, Canada, and New Brunswick, and from most of the States of this Union.

Of those on whom the degree of Doctor of Medicine has been this day conferred, seventy-two have spent one scholastic year in other incorporated institutions :—one in the Medical Department of Bowdoin College, Maine; two in that of Dartmouth College, New Hampshire; two in the Berkshire Medical Institution, Massachusetts; three in the Medical School of Castleton, Vermont; one in Geneva Medical College, New York; three in the University of New York; and two in the College of Physicians and Surgeons of that city; seven in the Medical Department of the University of Pennsylvania; and one in Pennsylvania Medical College; one in the Medical Department of the University of Maryland; and one in that of Washington University of the same state; six in that of Hampden Sidney College, at Richmond, Virginia; and fifteen in that of the University of Virginia; six in the Medical College of South Carolina; three in the Medical College of Georgia; eight in the Medical Department of the University of Louisville; and three in that of Transylvania University, Kentucky; four in the Medical College of Ohio; one in Willoughby Medical College; one in the Medical Department of the Western Reserve College, at Cleveland, Ohio; and one in the Medical College of Louisiana.

The afflux hither from other institutions must continue. It has been annually on the increase; and at no time, perhaps, has the ratio been as great as during the past session. The multiplication of medical schools, instead of diminishing the number of those that seek instruction in this city, augments it; for the facility of intercourse between the most distant places is so great, that a journey to Philadelphia is now within the means of a large proportion of medical students: hence it is, that so many visit her at least one winter, in order that they may enjoy those ample opportunities for full medical instruction, which have obtained for her the character of being the great centre of medical education on this side of the Atlantic."

